# Clinical Characteristics and Etiological Spectrum of Pancytopenia in Pediatric Age Group: A Cross-Sectional Outlook From a Developing Country

**DOI:** 10.7759/cureus.27842

**Published:** 2022-08-10

**Authors:** Abdullah Bin Zubair, Mustafa Tauseef Razzaq, Abdul Wasey Hashmi, Syed Muhammad Yasir Ali, Muhammad Muneeb Israr, Saad Mustafa Sadiq, Muhammad Fahad Khan, Zaki Haider, Muzammil Sabir, Mehwish Kaneez

**Affiliations:** 1 Research, Mayo Clinic, Rochester, USA; 2 Pediatrics, Rashid Latif Medical College, Lahore, PAK; 3 Internal Medicine, Shalamar Medical and Dental College, Lahore, PAK; 4 Pediatrics, Avicenna Medical College, Lahore, PAK; 5 Internal Medicine, Central Park Medical College, Lahore, PAK; 6 Pediatrics, Holy Family Hospital, Rawalpindi, PAK

**Keywords:** acute lymphoblastic leukemia (all), megaloblastic anemia, pediatric age group, bone marrow biopsy, pancytopenia

## Abstract

Background

The etiologies of pancytopenia in the pediatric age group remain exceedingly ubiquitous and warrant extensive hematological and interventional investigations like bone marrow biopsy. It varies widely from benign nutritional disorders to fatal malignancies. The present study aims to delineate the prevalence of various causes of pancytopenia in the pediatric population.

Methods

The present cross-sectional study included 96 patients between the age of one month till 15 years with pancytopenia. Study participants were evaluated for various parameters including their demographical details, clinical features, immunization history, and nature of the disorder. The prevalence of various etiologies (nutritional, neoplastic, infectious, autoimmune, and others) of pancytopenia was ascertained.

Results

Of the 96 patients, 42 (43.75%) were males with a mean age of 69.47 ± 7.12 months. Fever was present in 71.87%, arthralgias in 56.25%, weight loss in 35.41%, and failure to thrive in 18.75% of patients. The bone marrow examination revealed aplastic changes in 36 (37.50%), hyperplastic changes in 21 (21.87%), and normal cellularity in 40.62% of patients. Megaloblastic anemia was the most common nutritional cause of pancytopenia present in 21.85% of cases. Acute lymphoblastic leukemia (ALL) was the most prevalent neoplastic etiology present in 19.79% of patients. Aplastic anemia, miliary tuberculosis, parvovirus B19, and hemolytic anemia were other notable etiologies.

Conclusion

Megaloblastic anemia and infections like tuberculosis were common treatable etiologies of pancytopenia among the pediatric age group. ALL was the most common neoplastic etiology. Bone marrow biopsy remains crucial in elucidating the various neoplastic and nutritional etiologies of pancytopenia in children.

## Introduction

Pancytopenia is a common clinical entity that consists of a triad of anemia, leucopenia, and thrombocytopenia [1]. It results in a reduction in all cell lineages of bone marrow that leads to a vast array of clinical manifestations, especially among children [2]. These manifestations include (but are not limited to) fever, fatigue, dizziness, pallor, night sweats, anorexia, bleeding disorders, splenomegaly, hepatomegaly, and lymphadenopathy [3]. Pancytopenia is not a disease but a clinicopathological entity that can occur due to several disorders that primarily or secondarily affect the bone marrow [4]. Literature shows that pancytopenia affects nearly 3% to 5% of children; however, the data from developing countries regarding the etiological spectrum are scarce [5].

The etiological causes of pancytopenia range from non-malignant disorders to life-threatening malignant diseases like acute lymphoblastic leukemia (ALL) and acute myeloid leukemia. Some of these causes include aplastic anemia, nutritional deficiencies, myelodysplastic syndrome, leukemias, and autoimmune disorders [6]. Several infections including human immunodeficiency virus (HIV), miliary tuberculosis, leishmaniasis, and brucellosis can also cause pancytopenia among children [6-8]. Furthermore, some other factors like radiotherapy and chemotherapy can cause hypocellular bone marrow that leads to pancytopenia in children [5,9]. The spectrum of these etiologies is extremely varied and depends upon the geographical location, genetics, patient demographics, and nutritional profiles [10].

The initial investigation that is performed to confirm pancytopenia is the complete blood count (CBC). It will show hemoglobin (Hb) levels below 10 g/dL, platelet count lower than 100 × 10^3^/microL, total leukocyte count (TLC) under 4 × 10^9^/L, and absolute neutrophil count (ANC) below 1.5 × 10^9^/L [5]. Even though CBC is an important initial investigation, it cannot confirm the cause of pancytopenia. Therefore, a bone marrow biopsy and its examination under a microscope are considered a gold standard for the evaluation of its cause [5].

Pancytopenia in the pediatric age group has an extensive differential diagnosis; however, an undefined diagnostic approach makes it a huge challenge for pediatricians [2,3]. Moreover, morbidity and mortality of the affected children largely depend upon the disease progression, underlying pathology, and time of diagnosis [10]. All these factors necessitate the need for a profound understanding of the clinical characteristics and etiological profile of pancytopenia in children. Therefore, our study aims to assess and elaborate on the etiologies along with clinicopathological parameters of pancytopenia in the pediatric age group.

## Materials and methods

This cross-sectional study was conducted at the Department of Pediatrics, Holy Family Hospital, Rawalpindi, Pakistan. The study was conducted from September 2021 to May 2022. The study included a total of 96 patients aged between one month till 15 years with a confirmation of pancytopenia on CBC and a peripheral blood smear. The criteria were as follows: Hb levels below 10 g/dL, platelet count lower than 100 × 10^3^/microL, TLC under 4 × 10^9^/L, and ANC below 1.5 × 10^9^/L. A senior pediatrician took a complete clinical, sociodemographic, family, immunization, and drug history of all the patients. Thereafter, a physical examination was performed and the findings were noted. According to the findings of the history and physical examination, other relevant blood tests (serum iron levels, total iron-binding capacity, serum ferritin levels, vitamin B12 levels, coagulation screening, serum anti-HIV antibody levels, folate levels, detailed liver function tests, and Coomb’s test) were performed if required. In addition, radiological investigations like chest X-rays and abdominal ultrasounds were also performed as per indication and feasibility. Thereafter, bone marrow biopsies were performed for complete histopathological analysis of bone marrow. Patients with a diagnosis of cancer, recent blood transfusion history, and diagnosed cases of aplastic anemia were excluded from the study. Moreover, patients on chemotherapy or radiotherapy for ongoing treatment of a neoplastic disease were also subjected to exclusion. The ethical approval was obtained from the institutional review board of Holy Family Hospital with approval number IRB-2021-PM-023.

Patients were evaluated for multiple parameters including the demographical details, clinical features, diagnosis, and findings on bone marrow examination. Categorical variables such as gender, symptoms, diagnosis, and bone marrow examination findings were presented as frequency and percentages. Continuous data were presented as mean and standard deviation. Simple descriptive statistics were applied to report the various etiologies. Statistical Package for Social Sciences (SPSS) version 25.0 software (IBM Corporation, Armonk, NY, USA) was utilized for data entry and analysis.

## Results

In the current study involving 96 patients, the mean age of the study participants was 69.47 ± 7.12 months with a range between one month and 12 years. Fever was present in 71.87%, arthralgias in 56.25%, weight loss in 35.41%, and failure to thrive in 18.75% of patients. Most of the patients had multiple symptoms on presentation. The details of the study participants are delineated in Table 1.

**Table 1:** Baseline characteristics of the study participants.

Parameter		Frequency	Percentage
Gender	Male	42	43.75
	Female	54	56.25
Age groups	1 month till 1 year	13	13.54
	1-3 years	16	16.66
	3-5 years	28	29.17
	5-10 years	21	21.87
	More than 10 years	18	18.76
Immunization history	Complete immunization	44	45.84
	Partial immunization	31	32.29
	No immunization	21	21.87
Presenting symptoms	Fever	69	71.87
	Pallor	57	59.37
	Arthralgias	54	56.25
	Failure to thrive	18	18.75
	Weight loss	34	35.41
	Tremors	9	9.37
	Jaundice	8	8.33
	Vomiting	14	14.58
Clinical features	Hepatomegaly	51	53.12
	Splenomegaly	48	50
	Lymphadenopathy	16	16.66
	Hyperpigmentation	9	9.37
	Bleeding	38	39.58

In all patients, a bone marrow biopsy was performed for a definitive diagnosis of pancytopenia. The bone marrow examination revealed aplastic changes in 36 (37.50%), hyperplastic changes in 21 (21.87%), and normal cellularity in 39 (40.62%) patients. Megaloblastic anemia was the most common nutritional cause of pancytopenia present in 21.85% of cases. ALL was the most prevalent neoplastic etiology present in 19.79% of patients. Miliary tuberculosis was the most common infective etiology with 5.21%. Other notable etiologies were aplastic anemia, myelofibrosis, and sideroblastic anemia. There was a wide spectrum of neoplastic and non-neoplastic etiologies of pancytopenia. The spectrum of hematological diseases is elucidated in Table 2.

**Table 2:** A tabulation of hematological diseases and the associated prevalence in children with pancytopenia. HIV: human immunodeficiency virus; ALL: acute lymphoblastic leukemia; AML: acute myeloid leukemia.

Type of disorder	Diagnosis	Frequency	Percentage
Nutritional	Iron deficiency anemia	9	9.38
	Megaloblastic anemia	21	21.87
	Mixed deficiency anemia	6	6.26
Infective	Parvovirus B19	2	2.08
	Leishmaniasis	2	2.08
	HIV	1	1.04
	Miliary tuberculosis	5	5.21
	Salmonella species	1	1.04
	Chronic granulomatous disease	2	2.08
	Complicated malaria	2	2.08
Autoimmune	Systemic lupus erythematosus	2	2.08
	Immune thrombocytopenic purpura	1	1.04
	Hemolytic anemia	3	3.12
Neoplastic	ALL	19	19.80
	AML	6	6.26
Other	Aplastic anemia	7	7.30
	Myelofibrosis	2	2.08
	Hypoplastic bone marrow	3	3.12
	Sideroblastic anemia	2	2.08

## Discussion

Pancytopenia is a prevalent pathological finding that is frequently encountered in the pediatric age group. It has a multitude of underlying causes that determine the management and prognosis [11]. An appropriate clinical history, physical examination, laboratory investigations, and bone marrow examination are some necessary prerequisites for the assessment of the underlying etiology of pancytopenia [12]. Timely recognition of the underlying pathology can reduce mortality and morbidity. The male-to-female ratio in our study was 1:1.3. A similar study reported a ratio of 1:1.25 in their study with a non-significant female dominance [12]. In contrast, another study reported a male predominance [13]. These differences in gender predominance may be due to genetic differences, geographical variance, and disparity in nutritional status.

The most frequent presenting symptoms in children with pancytopenia were fever, pallor, arthralgias, and weight loss. Similar presenting complaints have also been reported in the literature [5,9,10]. These symptoms point toward a discrepancy in bone marrow cellularity that leads to a reduction in three of the cell lines. The low leukocytes make the patients prone to various infections causing fever and low Hb translates into symptoms like pallor and arthralgias [2]. Hepatomegaly, splenomegaly, lymphadenopathy, and bleeding were frequent clinical features that were assessed among patients. Similar features were reported in other studies from developing countries [10,14].

Megaloblastic anemia was reported to be the most common nutritional cause of pancytopenia among children in our study with a prevalence of 21.85%. Other studies from Pakistan and India also illustrated similar findings with a prevalence ranging between 10% and 36% [5,10,13]. Malnutrition in children, especially in developing countries, is the main cause of megaloblastic anemia that ultimately translates into pancytopenia [3]. Resource deprivation, unhealthy dietary habits, and poverty in developing countries also lead to other nutritional etiologies like iron deficiency anemia and mixed deficiency anemia [5]. Adequate delivery of vitamin B12-fortified food and supplementation of vitamin B12 and folic acid can help in reducing these causes.

Miliary tuberculosis was reported to be the most common infective cause of pancytopenia in our population. A significant portion of the population in developing countries like Pakistan is prone to develop tuberculosis due to poor immunization status and increased exposure [15]. Miliary tuberculosis can be a fatal infectious etiology that can be prevented with immunization and timely diagnosis and treatment with antituberculous drugs [9]. We also reported other etiologies like malaria, leishmaniasis, Salmonella, and HIV as infectious causes of pancytopenia. Other studies also reported malaria, visceral leishmaniasis, brucellosis, and sepsis as the common infections etiologies of pancytopenia [10,16]. This represents the wide spectrum of infectious etiologies of pancytopenia in children.

Additionally, neoplastic causes of pancytopenia in children cannot be overlooked. Even though neoplastic etiologies of pancytopenia in children are more common in developed countries, its prevalence in developing countries is also on the rise [9,17]. The present study delineated ALL to be a major neoplastic cause of pancytopenia. A study from India reported comparable findings with ALL being a common neoplastic cause of pancytopenia among the pediatric age group [18]. Other studies from developing countries report the prevalence of ALL to be between 13% and 23% [5,10]. A few other less prevalent causes of pancytopenia reported in our study were aplastic anemia, myelofibrosis, and sideroblastic anemia. Other studies have also reported similar patterns [4,5,10].

A limitation of our study is the inclusion of only one study center. A multicentric study from different hospitals would have enabled us to describe the prevalence of these etiologies in a precise manner. Furthermore, the non-inclusion of socio-economic and cultural parameters also accounts for a study limitation. Nonetheless, the results of our study necessitate the need for dietary modifications with iron and vitamin B12 supplementation in children living in developing countries so that nutritional causes of pancytopenia can be avoided. Moreover, the development of cancer screening programs and timely bone marrow examination in children with a suspected neoplastic cause can aid in their timely diagnosis and prevent mortality.

## Conclusions

There is a wide etiological spectrum of pancytopenia in the pediatric population that includes nutritional, infectious, autoimmune, and neoplastic etiologies. Of these, megaloblastic anemia remains the most common nutritional cause and miliary tuberculosis remains the most prevalent infectious cause. ALL is noted to be the most prevalent malignant cause of pancytopenia. Infectious causes of pancytopenia can be prevented with the improvement of immunization programs and empirical antibiotic therapy. The nutritional etiologies can be reduced with improved dietary habits, and vitamin B12 and iron supplementation.
